# Bacterial Toxins and Targeted Brain Therapy: New Insights from Cytotoxic Necrotizing Factor 1 (CNF1)

**DOI:** 10.3390/ijms19061632

**Published:** 2018-05-31

**Authors:** Elena Tantillo, Antonella Colistra, Eleonora Vannini, Chiara Cerri, Laura Pancrazi, Laura Baroncelli, Mario Costa, Matteo Caleo

**Affiliations:** 1 CNR Neuroscience Institute, via G. Moruzzi 1, 56124 Pisa, Italy; e.tantillo@fpscience.it (E.T.); anto_colistra@yahoo.it (A.C.); e.vannini@in.cnr.it (E.V.); c.cerri@in.cnr.it (C.C.); laura.pancrazi@sns.it (L.P.); baroncelli@in.cnr.it (L.B.); costa@in.cnr.it (M.C.); 2 Fondazione Pisana per la Scienza Onlus (FPS), via Ferruccio Giovannini 13, San Giuliano Terme, 56017 Pisa, Italy; 3 Departement of Biology, University of Pisa, via Luca Ghini 13, 56126 Pisa, Italy; 4 Fondazione Umberto Veronesi, Piazza Velasca 5, 20122 Milano, Italy

**Keywords:** CNF1, Rho GTPases, glioma, plasticity, cerebral cortex

## Abstract

Pathogenic bacteria produce toxins to promote host invasion and, therefore, their survival. The extreme potency and specificity of these toxins confer to this category of proteins an exceptionally strong potential for therapeutic exploitation. In this review, we deal with cytotoxic necrotizing factor (CNF1), a cytotoxin produced by *Escherichia coli* affecting fundamental cellular processes, including cytoskeletal dynamics, cell cycle progression, transcriptional regulation, cell survival and migration. First, we provide an overview of the mechanisms of action of CNF1 in target cells. Next, we focus on the potential use of CNF1 as a pharmacological treatment in central nervous system’s diseases. CNF1 appears to impact neuronal morphology, physiology, and plasticity and displays an antineoplastic activity on brain tumors. The ability to preserve neural functionality and, at the same time, to trigger senescence and death of proliferating glioma cells, makes CNF1 an encouraging new strategy for the treatment of brain tumors.

## 1. Introduction

Toxins represent potent weapons used by pathogenic bacteria to interact with target tissues of host organisms and to efficiently manipulate host cellular functions in a way that can favor survival and spreading of microbes. These molecules are responsible for a variety of severe disorders affecting animals and humans, including neurological, cardiovascular, enteric, but also multifactorial pathologies. While some toxins, indeed, display a targeted action on specific cell populations—such as enterotoxins affecting epithelial intestinal cells and neurotoxins corrupting neuronal cells—others cause the indiscriminate disruption of a wide range of cell types. This type of pathogenic proteins, called cytotoxins, affects the principal cellular regulators conditioning the main vital functions of eukaryotic organisms [1,2]. The modes of action that allow cellular aggression are various, but they may be classified as membrane damaging or intracellular acting toxins [3]. This is achieved by the recognition of specific cell surface receptors and/or intracellular targets, affecting vital processes of living cells. After binding to receptors on the plasma membranes of sensitive cells, toxins can either (i) interfere with signal transduction pathways, pore formation, or enzymatic activities at cell membrane; or (ii) translocate across the membrane barrier and modify specific intracellular targets, causing a dramatic alteration of cellular functions such as protein synthesis, cell homeostasis, cell cycle progression, vesicular traffic, and actin cytoskeletal rearrangements [1,4].

Since molecular mechanisms, cellular receptors, and structure of many toxins have been extensively studied, they are increasingly being considered as valuable tools for analysis of cellular physiology. Furthermore, it has become clear that the exquisite specificity of toxins can be exploited for the treatment and diagnosis of several human pathologies. Table 1 summarizes the potential exploitation of bacterial and scorpion toxins for the treatment of human diseases. One prototypical example are botulinum neurotoxins (BoNTs), a family of bacterial proteases which block neurotransmitter release by cleaving essential synaptic proteins. They are currently employed for the treatment of several disorders characterized by hyperexcitability of peripheral nerve terminals [5,6].

As a consequence, in recent years an increased number of toxin-based therapies arose to treat pain, inflammatory, and muscle disorders [7,8,9,10]. A current challenge is the study of bacterial toxins as possible drugs for treating brain pathologies such as neurodegenerative disorders and tumors.

Among brain tumors, glioblastoma multiforme (GBM) is the most aggressive form. Despite a variety of therapies (i.e., chemotherapy and radiotherapy) frequently associated to severe long-term side effects, the survival rate is only about 3–4% [11,12]. Thus, there is a strong need for developing more effective strategies to treat GBM. An important goal is the validation of treatments that are less invasive for the peritumoral healthy brain tissue. In this framework, toxins represent a promising tool for GBM therapy because of their features in terms of specificity and biological action. In particular, (i) toxins are extremely potent and not subjected to mechanisms of drug resistance; (ii) the active core of the toxins can be isolated and cloned becoming small in molecular size, and thus more efficient to penetrate into solid tumors; (iii) toxins can be combined by recombinant DNA technology with selective carriers or antibodies (Abs) against surface receptors, thus allowing specific entry of the catalytic toxin domain into selected tumor cell types [13].

Cytotoxic necrotizing factor 1 (CNF1) is one of the virulence factors produced by some pathological strains of extraintestinal *Escherichia coli.* CNF1 exerts its action on Rho GTPases, thus impairing hydrolysis of guanosine triphosphate (GTP) inducing a long-lasting activation of such proteins with a consequent impact on fundamental cellular processes [14]. Recent studies performed in our lab demonstrated anti-neoplastic effects of CNF1 in a mouse model of glioma. Indeed, we found that CNF1 has an important double action: (i) it is detrimental for tumoral cells, since it is able to block cytodieresis in proliferating cells, leading them to senescence and death; (ii) it possesses a beneficial activity on peritumoral neurons, enhancing their plasticity and maintaining their functional properties.

In this review, we deal with the possibility of exploiting this toxin as a “plasticizing” and antineoplastic agent in the neural tissue, highlighting the potential therapeutic relevance of CNF1 in the treatment of brain tumors. We also review the potential use of CNF1 in neurodegenerative pathologies with altered Rho GTPases signaling and impaired neural plasticity, such as Rett syndrome, Alzheimer’s, and Parkinson’s disease [15,16,17,18].

## 2. Cytotoxic Necrotizing Factor (CNF1) Structure and Mechanism of Action

**Figure 1:**
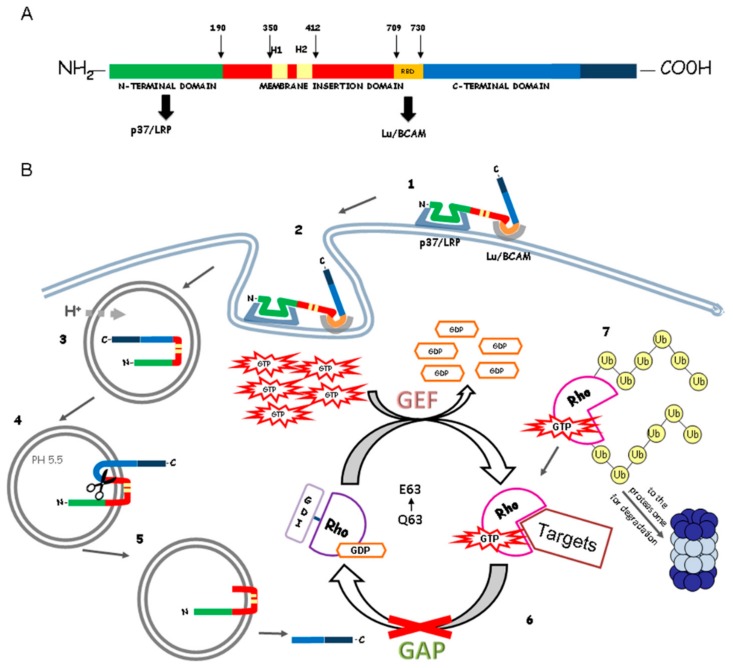
Structure and mechanisms of action of cytotoxic necrotizing factor (CNF1). (**A**) Molecular structure of the *Escherichia coli* CNF1. The toxin domains are represented with different colors and are delimited by numbers (located above the schematic structure), which denote the amino acid residues. The black arrows below the structure indicate the cell-binding domains for p37/LRP and Lu/BCAM receptors. The two yellow boxes (H1 and H2) are the two hydrophobic helices; (**B**) Mechanisms of CNF1 cell entry in target cells and modulation of Rho GTPase activation. Once in the cytosol, the catalytic domain of CNF1 catalyzes the deamidation of a specific glutamine residue (Q63), which is converted to glutamate (E63). GDI, GDP-dissociation inhibitor; GEF, guanine exchange factor; GAP, GTPase activating protein; Ub, ubiquitin.

Cytotoxic necrotizing factor 1 (CNF1) is a 114 kDa single-chain bacterial protein toxin which exerts a very specific control of Rho GTPase activity. It is composed of three domains (see Figure 1A): the N-terminal domain, the translocation domain and the C-terminal domain, indispensable for its catalytic activity [3,19]. CNF1 was first described in 1983 by Caprioli and co-workers as a toxin capable of causing multinucleation (“cytotoxic”) in cultured cells and necrosis in rabbit skin (“necrotizing”) [20]. While the necrosis was caused by a contamination in toxin preparation, the ability of multinucleating proliferating cells is a specific action of CNF1. The mechanisms of CNF1 action on target cells are summarized in Figure 1B.

It has recently been discovered that CNF1 can bind two different receptors. The p37/ laminin receptor precursor (p37/LRP), crucial for CNF1 action, and the Lutheran adhesion glycoprotein/basal cell adhesion molecule (Lu/BCAM), necessary for the binding between toxin and cells. The toxin enters mammalian cells by receptor-mediated, clathrin-independent endocytosis: CNF1 binds to laminin receptor 67LR and its precursor p37/LRP through the N-terminus (aa 1–342) and to Lu/BCAM through a region located close to the C-terminal catalytic domain [21,22]. CNF1 N-terminal portion has two cell-interaction sites and, after binding to its receptors, enters endocytic vesicles by receptor-mediated endocytosis; progressively, the toxin is routed to the endosomal compartment and the catalytic domain is transferred into the cytosol [23]. The CNF1 translocation domain presents two helices (H1 and H2) separated by a hydrophilic loop, which is thought to be essential for the membrane collocation [24]. It has been demonstrated that a toxin fragment of approximately 55-kDa containing the catalytic domain and an additional part is present in the cytosol (Figure 1B). Its processing requires an acidic pH (≤5.2) and the insertion of the toxin into the endosomal membrane [19]. The cleavage site of CNF1 is located between amino acids 536 and 542. Experiments with CNF1 mutants have shown that the processing and release of the catalytic part from the endosomes is essential for the full biological activity of CNF1. Presumably, this release occurs from late endosomes, because the destruction of the microtubules, necessary for the maturation from early to late endosomes, results in weaker CNF1 toxicity [23,25].

The cytoplasmic targets of CNF1 are the Rho GTPases, a family of molecular switches that cycle between a Guanosine diphosphate (GDP) -bound inactive to a GTP-bound active state [26,27]. Once in the cytosol, CNF1 catalyzes the deamination of glutamine 63 in RhoA and glutamine 61 in Rac and Cdc42, blocking the hydrolysis of GTP and leaving Rho GTPases in a permanent activated state [28,29]. This is followed by partial deactivation of Rho GTPases via degradation by the ubiquitin-proteasome pathway [30]. CNF1-induced Rho GTPase activation is sufficient to trigger a massive reorganization of the actin cytoskeleton, which becomes unable to orchestrate cytodieresis, despite ongoing nuclear division. As a consequence, proliferating cells exposed to CNF1 display a multinucleated phenotype [20]. Moreover, CNF1-dependent activation of Rac results in the degradation of the nuclear factor (NF-κB) inhibitor IkBα and NF-κB nuclear translocation [31], leading to the stimulation of DNA transcription and the expression of pro-inflammatory factors [32] that can protect host cell from apoptotic stimuli [33].

## 3. Effects of CNF1 on Neurons

Rho GTPases control a wide variety of signal transduction pathways in all eukaryotic cells including gene transcription, actin cytoskeleton organization, cell proliferation, and survival [34]. Importantly, Rho GTPases are key regulators of actin polymerization, and are therefore involved in many developmental processes that require cell morphological changes, like neuronal migration, dendrite, and axon growth and guidance of neural cells. Specifically, Rac1 and Cdc42 are required for neurite formation and outgrowth; conversely, Rho activation suppresses neurite outgrowth inducing their retraction [35,36].

By activating Rho GTPases, CNF1 induces a remarkable reorganization of the actin cytoskeleton [3]. This capability, previously observed in epithelial cells [37,38], has also been demonstrated in neurons [39,40,41,42]. Indeed, intracerebral injection of CNF1 in adult rodents leads to a long-lasting activation of Rac1 that results in marked neural structural remodeling. In particular, we demonstrated that a single CNF1 injection in rat visual cortex significantly increases spine density and length in pyramidal neurons, with no deleterious effect on neuronal survival [40]. Accordingly, both chronic in vivo two-photon imaging and Golgi staining reveal that intracerebroventricular injection of CNF1 increases spine density in visual and hippocampal neurons, albeit with region-specific features. Indeed, structural changes are evident in both apical and basal dendrites in hippocampal pyramidal neurons, while in primary visual cortex they are restricted to basal dendrites [41]. Results from in vitro experiments suggest that these morphological changes are probably mediated by astrocytes, as direct administration of CNF1 to neuronal cultures has a harmful effect on neuronal maturation, while hippocampal neurons grown in CNF1-treated astrocytes show an increased development of neurites [42]. However, the mechanisms by which CNF1 modulates neuronal structure via astrocytes remain to be clarified.

CNF1-induced neuronal remodeling has important functional consequences. In vitro experiments have shown that CNF1 enhances glutamatergic neurotransmission and long-term potentiation in hippocampal neurons [39,41]. Noteworthy, we demonstrated that in vivo stimulation of structural rearrangements by CNF1 is able to reinstate functional plasticity in the adult rat visual cortex. Indeed, adult rats treated with CNF1 show an ocular dominance (OD) shift toward the open eye after monocular deprivation (MD), a classical test for adult cortical plasticity [36]. CNF1-mediated OD plasticity is selectively attributable to the potentiation of open-eye responses, an effect that correlates with increased density of geniculocortical terminals in layer IV of monocularly deprived CNF1-treated rats [40]. This boost of neural plasticity is likely to be one of the mechanisms underlying the improvement of cognitive functions induced by CNF1. Indeed, CNF1 treated mice display improved learning and memory in various behavioral tasks [39,43].

It has been reported that peripheral or central administration of CNF1 has an analgesic effect in formalin-induced inflammatory pain in mice, due to induced upregulation of μ-opioid receptors (MORs), the most important receptors controlling pain perception. CNF1 effects on inflammatory pain have been associated with sustained Rac activation and the consequent actin cytoskeleton rearrangement [7]. However, the potential pharmacological relevance of CNF1 is particularly evident in those pathologies where Rho GTPases signaling and spine morphology appear to be consistently affected. Several preclinical studies on Rett Syndrome (RTT) demonstrated [15,44,45] that CNF1 rescues cognitive deficits, aberrant synaptic plasticity, astrocytes defects, and mitochondrial dysfunction in the MeCP2-308 RTT mouse model through the modulation of brain Rho GTPases that appear to be involved in RTT pathophysiology [29,30,31,32,33].

Similarly, it has been found that CNF1 improves memory performance, decreases β-amyloid accumulation and controls neuroinflammation in a mouse model of Alzheimer’s disease [17].

In Parkinson’s disease (PD), in which derangement of Rho GTPase signaling has been observed, delivery of the toxin has been tested as a potential neurorestorative approach. The data indicate that CNF1 displays neurotrophic effects on dopaminergic neurons both in vitro and in vivo [18].

It has also been demonstrated that a single injection of CNF1 in tDBA/2J mice (an animal model with high susceptibility to induced- and spontaneous epileptic seizures) is able to induce a remarkable amelioration of the seizure phenotype, increasing markers of neuroplasticity and mitochondrial ATP, necessary to decrease seizure generation [46]. Altogether, these findings highlight the great potential of CNF1 as therapeutic tool for a broad range of brain pathologies.

## 4. Effects of CNF1 on Cancer Cells

The permanent activation of Rho GTPases proteins by CNF1 affects the regulation of different cellular processes fundamental for the cell fate. The persistent activity of Rho GTPases impacts on pathways whose dysregulation is mainly involved in cancer progression, such as actin cytoskeleton organization, cell division, migration, and survival [47]. Therefore, it is interesting to investigate the effect that CNF1 can exert on the main events of tumor development. Two crucial aspects of that process are represented by apoptosis and the regulation of actin cytoskeleton [48]. The effect of CNF1 on apoptosis is still debated. The toxin appears to protect epithelial cells from apoptosis induced by exposure to ultraviolet-B (UVB) irradiation [45]. In particular, proteasomal degradation of the Rho GTPase is necessary to achieve cell death protection, because inhibition of Rho degradation abolishes the pro-survival activity of CNF1. An intriguing hypothesis is that Rho inactivation provokes a dominant activity of Rac [49], as it is well known that Rho family members antagonize each other [50]. In addition, the activation of the Phosphatidylinositol 3-kinase (PI3K)/serine-threonine protein kinase AKT/Inhibitor of kB kinase (IKK)/NF-κB pathway leads to the transcription of the antiapoptotic factor Bcl-2, which is responsible of CNF1 pro-survival effect in UVB-irradiated Hep-2 cells. CNF1 provokes a transient RhoA activation in some androgen dependent cell lines, such as LNCaP cells, that are not strong enough to induce cell death [48]. On the other hand, it has been described that CNF1 can induce apoptosis in 5637 bladder carcinoma cells by stimulating the secretions of cytokines involved in the inflammatory response, TNF-α, and factors for the neutrophilic response [51]. Moreover, it has been clearly demonstrated that the activation of Rho GTPases is necessary for an apoptotic response in prostate cancer cells [52,53].

Rho GTPases are the leading proteins in the organization of the actin cytoskeleton [54,55]. Their continuous activation improves cell motility and invasiveness in uroephitelial 804 G cells [48,56,57], the migration and metastasis in prostate cancer cell lines [58], nuclear segmentation, and macropinocytosis [59]. In several cell lines, the alteration of actin-dependent events induced by CNF1 prevents a correct cell division process. The effect of CNF1 on actin and tubulin organization is well described on Hep-2 epithelial cells, in which the toxin induces membrane ruffles at the cell border and thick bundles of actin crossing the cell body. This interferes with cytokinesis, blocking the cytodieresis, while nuclear division happens with the consequent multinucleation [20,37,60]. The activation of the Rho GTPases plays a pivotal role also in the regulation of the cell cycle. Indeed, in the uroepithelial T24 cell line, CNF1 induces the down regulation of the cyclin B1 expression and its elimination from the cytoplasm causes a block of the tumor cells in the G2/M phase without triggering an apoptotic response [27] as demonstrated for HeLa cells [61].

Thus, CNF1 is a protein with a very complex spectrum of activities that could be dependent upon cell microenvironment, cell type, and condition [56]. Despite results supporting the protumoral activity of CNF1 in some conditions, the toxin is also able to induce apoptosis, to arrest cell proliferation in tumor cells and to induce multinucleation. These results, together with the effect of protecting neuronal structure and function in health and disease [18,39,40], suggest that CNF1 may be tested as a potential anti-neoplastic agent in brain tumors. The toxin could represent a tool to manipulate specific cellular pathways involved in cancer progression, blocking tumoral growth but also preserving the surrounding, healthy tissue.

## 5. CNF1 Action in Glioma: Functional Sparing of Peritumoral Neurons

Recently, we provided strong evidence that CNF1 represents a promising drug for the treatment of glioma. Gliomas are primary central nervous system tumors that arise from astrocytes, oligodendrocytes or their precursors. Glioblastoma multiforme (GBM; median survival expectancy of 15–18 months after diagnosis) represents the most malignant form. The standard-of-care for GBM consists in surgical resection of tumoral mass followed by cycles of chemo- and radiotherapy. Unfortunately, a cure for this disease is still lacking [12].

**Figure 2:**
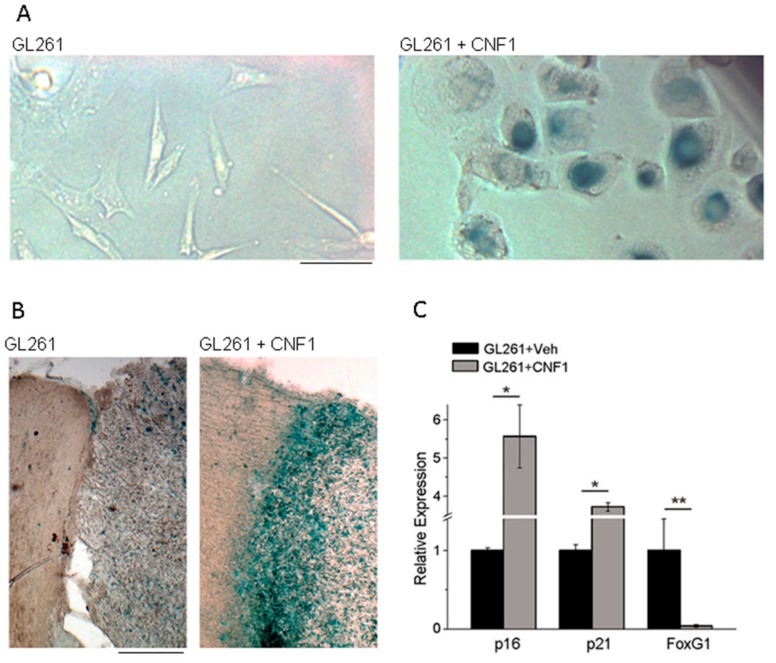
CNF1 treatment triggers senescence of glioma cells. (**A**) β-galactosidase staining (blue; a marker for senescence) in cultured GL261 glioma cells treated with either vehicle (**left**) or CNF1 (**right**). Note multinucleated, senescent cells after CNF1 treatment; (**B**) Representative brain sections from glioma-bearing mice treated with either vehicle (**left**) or CNF1 (**right**). Note robust staining for β-galactosidase (blue) in the CNF1-treated sample. Scale bar = 10 µm for (**A**), 100 µm for (**B**); (**C**) Quantitative real-time PCRs showing the relative expressions of the senescence markers p21 (Cdkn1a, Cyclin-dependent kinase inhibitor 1) and p16 (Cdkn2a, Cyclin-dependent kinase inhibitor 2), and the negative p21 regulator FoxG1 (Forkhead box protein G1) in GL261 cells treated with CNF1 or vehicle. * *p* < 0.05, ** *p* < 0.01. Panel B modified and C taken from Vannini et al., 2016 [63].

We found that CNF1 impairs motility and proliferation of both murine and human glioma cells causing their death in 15 days [62,63]. In vitro and in vivo assays showed that CNF1 triggers molecular and morphological hallmarks of senescence (Figure 2), which eventually lead murine and human glioma cells to death. Specifically, CNF1-treated glioma cells show an overexpression of p21 and p16, while *FoxG1* is downregulated; these markers point to an activation of the senescence process, that was also confirmed by β-galactosidase staining (Figure 2) [62,63].

Moreover, in vivo analysis revealed that glioma-bearing mice treated with a single, low dose of CNF1 (2 nM) showed a significantly extended survival which is comparable to that obtained with a prolonged treatment with temozolomide (TMZ; 140 µM), a widely-used chemoterapic agent. Increasing the CNF1 concentration up to 80 nM leads to a dramatic increase in the survival of glioma-bearing mice with 57% of animals surviving up to 60 days following glioma cell transplant [62]. Consistently, CNF1-treated glioma-bearing mice revealed a strongly reduced tumoral volume when compared to both TMZ- and vehicle-treated animals [63,64].

**Figure 3:**
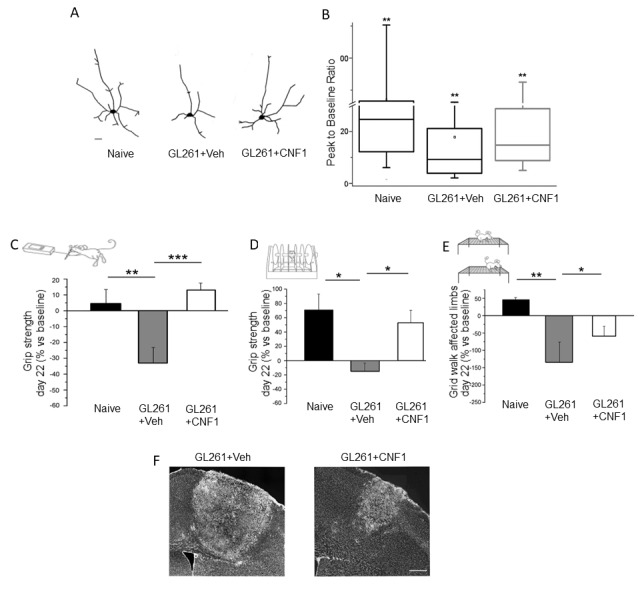
CNF1 treatment spares the morphological and functional properties of peritumoral neurons. (**A**) Representative reconstructions of layer V pyramidal neurons in naïve mice, and glioma-bearing mice treated with either vehicle or CNF1. Scale bar = 10 µm; (**B**) CNF1 treatment partly prevents the reduction of visual responsiveness in peritumoral neurons. GL261 cells were implanted in the mouse visual cortex and electrophysiological recordings were performed in the peritumoral areas. Responsiveness was quantified as the peak response to the visual stimulus divided by spontaneous activity (peak-to-baseline ratio); (**C**–**E**) Quantification of motor deficits in the grip strength (**C**), rotarod (**D**), and gridwalk tests (**E**) following experimental induction of glioma in the mouse motor cortex. Performances are measured in baseline (before glioma induction) and then longitudinally along disease progression until day 22. Note that CNF1 treatment maintains motor fufinanction in glioma-bearing mice; (**F**) CNF1 treatment reduces tumor volumes, as shown by representative brain sections containing the tumoral mass (bright labelling). Scale bar = 150 µm. * *p* < 0.05, ** *p* < 0.01, *** *p* < 0.001. Panels taken from Vannini et al., 2016 [63] and Vannini et al., 2017 [64].

However, innovative and efficient approaches for the treatment of glioma patients should aim not only at targeting glioma growth, but also at preventing functional deterioration of spared brain networks, preserving the surrounding, peritumoral, healthy tissue. Accordingly, we performed—for the first time in literature—electrophysiological and behavioral studies on glioma-bearing mice to assess functional sparing of peritumoral areas. The data showed that, compared to naïve animals, glioma-bearing mice display shrunken peritumoral neurons with reduced dendritic branching and impaired neuronal responsiveness [63]. All these alterations were clearly induced by tumoral growth but, interestingly, could be counteracted by CNF1 treatment. Indeed, CNF1-treated, glioma-bearing animals exhibited a partially protected neuronal dendritic branching and architecture, together with maintained physiological properties of pyramidal neurons [63]. Remarkably, the treated mice displayed electrophysiological and behavioral parameters that were significantly spared as compared to control glioma-bearing animals [63,64]. In a recent experiment, glioma-induced dysfunction was longitudinally monitored via behavioral analyses of motor function. The findings showed that CNF1, when delivered intracerebrally at a symptomatic stage (i.e., after the appearance of motor deficits), was remarkably effective in protecting from functional deterioration and in reducing tumor volumes (Figure 3) [64].

Altogether, these data indicate that CNF1 delivery represents a novel and very promising strategy for glioma therapy. Indeed, beyond blocking tumor progression and migration, this bacterial toxin is capable of preserving the healthy surrounding tissue, protecting its architecture and functionality.

## 6. Concluding Remarks

In this review, we have described the potential therapeutic relevance of CNF1 and discussed its possible applications in the field of brain tumors.

The ability of protein toxins to modulate fundamental cell functions in a very specific manner makes them ideal candidates to be employed for therapeutic applications. Therefore, a variety of bacterial toxins (either native or conjugated to antibodies and other drugs) are currently being tested for medical purposes in order to find new strategies to counteract brain pathologies such as neurodegenerative disorders and tumors.

Among the potentially useful toxins for the treatment of brain tumors, CNF1 represents an ideal candidate. CNF1 exert its specific activity as a constitutive activator of Rho GTPases proteins such as RhoA, Rac, and Cdc42 [26,27,28,29] by the action of its cytosolic catalytic domain [23], released from endosomes after receptor-mediated clathrin-independent endocytosis [21,22]. This specific interaction affects a wide variety of cellular pathways, and, mainly, the actin cytoskeleton organization [34]. For this reason, CNF1 could be exploited as a therapeutic agent for those pathologies in which morphological changes occur during neural developmental processes. Consistently, our findings demonstrate a plasticizing effect on neural structures induced by CNF1 after cerebral injection in rodents. In particular, together with the increase of spine density and the enhancement of functional plasticity, we have shown the absence of deleterious effects on neuronal survival after CNF1 intracerebral delivery, an indispensable feature that makes this toxin a good candidate for neurological applications [24,25]. It is important to mention, however, that at least one population of central neurons—i.e., retinal photoreceptors—were found to degenerate within a few days after CNF1 exposure [65]. In this study, CNF1-treated retinas showed high levels of reactive oxygen species. This could explain the selective vulnerability of photoreceptors, as they are known to be highly vulnerable to photo-oxidative stress.

Intriguingly, it has been demonstrated that the rearrangement of actin cytoskeleton induced by CNF1 has an analgesic effect in inflammatory pain [7] and a therapeutic effect on neurodegenerative disorders in which Rho GTPases activity seems to be involved such as Rett syndrome [15,44,45], and Alzheimer’s [17] and Parkinson’s [18] diseases. The ability of CNF1 to modulate and combine different specific cellular functions—such as the promotion of cell death through apoptosis or senescence [52,53], the arrest of cell proliferation [27], and the induction of multinucleation [20,37,60]—together with the effect of protecting neuronal structure and function highlights CNF1 as a novel therapy able to arrest cancer cell development in the central nervous system. However, its effect on apoptosis is still debated for a large variety of tumors, because the activation of the wide range of Rho GTPases, in some contexts, seems to be involved in cancer progression, invasion and metastasis protecting from cell death [66,67]. In this context, our group assessed in vitro and in vivo the potential anti-neoplastic activity. In cell culture, we demonstrated that CNF1 is able to impair motility and proliferation of both murine and human glioma cells that acquire a senescent phenotype after toxin administration. In the same experiments, we showed that a CNF1-based therapy can extend the survival of glioma-bearing mice in a manner which is comparable to a prolonged treatment with temozolomide (TMZ), reducing tumor expansion and, more importantly, maintaining the normal physiological properties of peritumoral tissue [62,63]. Altogether, these data strengthen the notion that CNF1 represents an innovative tool to block glioma development, protecting the not yet damaged surrounding tissues and maintaining the normal neural physiology. On the other hand, currently there are hurdles to the exploitation of this toxin for glioma therapy. One limitation is represented by the presence of the blood–brain barrier and the potential for side effects after systemic CNF1 administration. For these reasons, in the mouse studies conducted so far, the toxin was injected via an invasive intracerebral route. Second, the wide range of Rho GTPases on which the toxin can act could represent a limit, thus restricting clinical applications. To overcome this limit, the specificity of the target(s) seems to be crucial. For example, it has been discovered that the selective activation of RhoA/B/C by Yersinia cytotoxic necrotizing factor (CNFy) can stimulate the apoptotic pathway in specific tumor cells in which CNF1 has no effect [48]. Another alternative could be represented by immunotoxins, in order to precisely select the target cells through the specificity of an antibody [13,68]. Therefore, the use of more selective toxins might be a feasible new avenue for tumor treatments.

## Figures and Tables

**Table 1:** Current and potential therapeutic applications of protein toxins.

Toxin	Therapeutic Application
Botulinum neurotoxin (BoNT) from *C. botulinum*	Dystonia Muscle tone disorders Autonomic disorders Cosmetic use Pain therapy
Lethal toxin (LF) from *B. anthracis*	Potential treatment of cancer
Pertussis toxin (PTX) from *B. pertussis*	Potential use in control of HIV replication
Cytotoxic nectorizing factor 1 (CNF1) from *E. coli*	Potential use in learning and memory enhancement Potential treatment for neurodegenerative disorders Potential treatment of primary brain tumors
Immunotoxins	Cancer therapy
Chlorotoxins from *Leiurus quinquestriatus scorpion venom*	Potential treatment of primary brain tumors, currently used to deliver anti-cancer drugs specifically to cancer cells
